# Correction: Kim et al. Correlative Light and Electron Microscopy Using Frozen Section Obtained Using Cryo-Ultramicrotomy. *Int. J. Mol. Sci.* 2021, *22*, 4273

**DOI:** 10.3390/ijms23126440

**Published:** 2022-06-09

**Authors:** Hong-Lim Kim, Tae-Ryong Riew, Jieun Park, Youngchun Lee, In-Beom Kim

**Affiliations:** 1 Integrative Research Support Center, College of Medicine, The Catholic University of Korea, Seoul 06591, Korea; wgwkim@catholic.ac.kr (H.-L.K.); bloomwood@catholic.ac.kr (J.P.); leeycf@catholic.ac.kr (Y.L.); 2 Department of Anatomy, College of Medicine, The Catholic University of Korea, Seoul 06591, Korea; trriew@gmail.com; 3 Catholic Neuroscience Institute, College of Medicine, The Catholic University of Korea, Seoul 06591, Korea; 4 Catholic Institute for Applied Anatomy, College of Medicine, The Catholic University of Korea, Seoul 06591, Korea

The authors wish to make a correction to this paper [1]. We inadvertently failed to mention an important previous work in the field. We would like to add the following sentence and a reference in the Discussion section on page seven, lines nine–ten, in-between these two sentences: “Pre-embedding immunogold labeling using 10-μm-thick frozen sections, which are obtained using a histo-cryostat, has been reported previously [13,14]” and “We compared the OCT-embedded histo-cryostat samples cut at −20 °C with the 2.3 M sucrose-embedded samples cut at −100 °C, and we found that the embedding media and cutting temperature are critical factors in reducing the ice crystal-induced cryo-damage”.

“Additionally, Kusumi et al. introduced CLEM using semithin (1 μm) cryosections at −65 to −75 °C in the backscattered electron (BSE)-mode scanning electron microscope [2].”

Newly added reference as [15] in original paper, the order of some reference citations has been adjusted correspondingly.

The authors apologize for any inconvenience caused and state that the scientific conclusions are unaffected. This correction was approved by the Academic Editor. The original publication has also been updated.
